# A systematic review protocol of injuries and illness across all the competitive cycling disciplines, including track cycling, mountain biking, road cycling, time trial, cyclocross, gravel cycling, BMX freestyle, BMX racing, e-sport, para-cycling and artistic cycling

**DOI:** 10.3389/fspor.2024.1385832

**Published:** 2024-10-25

**Authors:** Thomas Fallon, Neil Heron

**Affiliations:** ^1^ Centre for Public Health, Queen’s University Belfast, Belfast, Northern Ireland; ^2^ Edinburgh Sports Medicine Research Network & UK Collaborating Centre on Injury and Illness Prevention in Sport (UKCCIIS), Edinburgh, United Kingdom; ^3^ School of Medicine, Keele University, Staffordshire, England

**Keywords:** sport, participation, elite athletes, injury, illness, cycling, systematic reviews, protocol

## Abstract

**Introduction:**

The sport of cycling has witnessed phenomenal growth over the past decade. Globally, over 200 million television hours across five continents watched the recent inaugural World Championships in Glasgow, in 2023. The Union Cycliste Internationale (UCI), the world cycling governing body, has highlighted its mission to “*promote and support research in cycling epidemiology and medicine, especially for the benefit of lesser-known disciplines”* within its 2030 Agenda. This paper outlines a proposed protocol to conduct a systematic review that comprehensively analyses and synthesises the existing literature about cycling-related injuries and illness across all competitive disciplines.

**Methods:**

The Preferred Reporting Items for Systematic Reviews and Meta-analyses (PRISMA) guidelines will be followed through each stage of this systematic review. Cycling is an umbrella term used for many individual disciplines. Investigation of all types of injuries and/or illnesses sustained during training and/or competition among competitive athletes across all disciplines will be included in this review. A computerised, systematic literature search will be conducted in electronic databases MEDLINE, Scopus, Embase, and Cochrane Library. Independent screening by two reviewers in a two-step process: title/abstract screening followed by full-text review. The reference lists of included articles will be searched to identify any other potentially relevant articles. Narrative synthesis and tabular/charted presentation of the extracted data will be included.

**Discussion:**

This protocol paper outlines the methodology to conduct a systematic review of injuries and illness across all competitive cycling disciplines. The aims of outlining this systematic review protocol are to aid research transparency, help reduce publication bias, prevent selective publication, and prevent the selective reporting of results. Future systematic reviews based on the proposed protocol will summarise the known prevalence, incidences, locations and burden of injury and illness across the sport of cycling.

**Trial Registration:**

This study has been registered with the PROSPERO International Prospective Register of Systematic Reviews (registration number CRD42024502703).

## What is already known on this topic

• There is a lack of injury and illness epidemiology research within competitive cycling.
• In epidemiological studies completed to date, there is a lack of methodology homogeneity around the definitions of injury and illness and reporting standards.
• The Union Cycliste Internationale (UCI) Agenda 2030 acknowledges the lack of epidemiological research within cycling. It aims to “*promote and support research in cycling epidemiology and medicine, especially for the benefit of lesser-known disciplines”.*

## What this study adds

• This manuscript outlines a protocol that future studies can use to carry out a multidiscipline systematic review of injuries and illness in competitive cycling.

## How this study might affect research, practice, or policy

• This protocol can be repeated over time to give an up-to-date understanding of the prevalence of injury and illness within cycling across all competitive disciplines.
• Upon completion, studies that follow this proposed protocol will formally highlight the shortcomings in research and provide direction for further research within cycling.

## Introduction

Cycling is a popular and diverse sport, encompassing various disciplines, each with its distinctive demands, environments, and risk factors. Within the sport of cycling, there are ten broad cycling disciplines which break down into 40 subdisciplines of cycling. Globally, over 200 million television hours across five continents were broadcasted during the recent inaugural World Championships in Glasgow 2023 (1). The sport's phenomenal growth over the past decade has led to an increased focus on the occurrence, patterns and impacts of injuries and illness within different cycling disciplines (2, 3). Understanding the prevalence and nature of these injuries and illnesses is imperative for enhancing safety measures, injury prevention, and athlete well-being. However, epidemiology research within competitive cycling to date is scarce and lacks methodological homogeneity between studies and disciplines (4).

The Union Cycliste Internationale (UCI), which is the world governing body for cycling, oversees the regulation of each competitive cycling discipline and the growth of the sport. The UCI has over 1 million members across five continents and holds over 33,000 events per year (5). As per the 2030 UCI agenda, they highlight the drive to “*promote and support research in cycling epidemiology and medicine, especially for the benefit of lesser-known disciplines (epidemiology of medical and trauma pathologies*)” (p106) (5). To date, there has been limited endorsement of such initiatives by the UCI within competitive cycling, which challenges our ability as clinical academics to establish evidence-based injury prevention recommendations and programs.

With the diversity of athletes competing within competitive cycling, across a variety of disciplines, combined with the lack of epidemiological studies, it is unknown what the true prevalence and incidence of injury and/or illness within each discipline is. Indeed, to date, many disciplines have not even been represented within epidemiological research (4). The International Olympic Committee (IOC) published the first consensus extension for the reporting of injuries within competitive cycling in 2021 (4). Both the UCI Agenda 2030 (5) and the IOC consensus (4) called for action for epidemiology research in the sport, with a particular focus on the disciplines with no research to date.

The preliminary model underpinning injury prevention was proposed by Van Mechelen et al. in 1992 (6) in a 4-step model starting with injury surveillance. This was further revised by Finch in 2006 with the addition of 2 further steps and the formation of a model known as the TRIPP (Translating Injury Prevention to Practice) model (Table 1) (7). Both models highlight the importance of injury and illness surveillance as the foundation for developing evidence-based injury prevention programmes.

**Table 1:** Translating research into the injury prevention practice (TRIPP) framework (7).

Model Stage Translating Research into the Injury Prevention Practice Framework
1 Injury surveillance
2 Establish aetiology and mechanisms of injury
3 Develop preventive measures.
4 “Ideal conditions”/scientific evaluation
5 Describe the intervention context to inform implementation strategies
6 Evaluate the effectiveness of preventive measures in the implementation context

Future studies based on this proposed protocol will enable researchers to uncover commonalities, disparities, and critical gaps in the current knowledge of cycling-related injury and illness epidemiology within each discipline. This paper outlines the methods to conduct a systematic review that comprehensively analyses and synthesizes the existing literature about cycling-related injuries and illness across all competitive disciplines. While individual studies often concentrate on specific disciplines or specific injuries, this review seeks to collate and critically evaluate injury and illness research across all disciplines, including within para-cycling. Completing such research, will act as a foundation in our understanding of where the gaps lie in knowledge and focus our attention on better understanding and preventing injuries and illnesses in competitive cycling (6).

### Aim

To outline a protocol that will enable researchers to identify the prevalence, and incidence of injuries across each of the competitive cycling disciplines which include, track cycling, mountain biking, road cycling, time trial, cyclocross, gravel cycling, BMX freestyle, BMX racing, e-sport, para-cycling and artistic cycling.

### Objectives

1. To assess the epidemiology of injury across all cycling disciplines.
2. To assess the epidemiology of illness across all cycling disciplines.
3. To identify the differences in definitions and reporting methods of injuries and illnesses across all the cycling disciplines.

## Methods

### Study design

Systematic reviews based on the proposed protocol will follow a systematic review study design. The Preferred Reporting Items for Systematic Reviews and Meta-analyses (PRISMA) guidelines will be followed through each stage of this systematic review (8). Additionally, the PERSiST (implementing Prisma in Exercise, Rehabilitation, Sports Medicine, and SporTs science) document will be used as a guidance tool in developing this protocol and outcomes of focus (Appendix 2) (9).

### Study registration

The protocol for this systematic review has been registered on the PROSPERO international prospective register for systematic reviews with registration number CRD42024502703.

### Ethics and dissemination

Ethical approval is not required for systematic review studies that follow this protocol. The intention is to disseminate the findings through a peer-reviewed journal and present them at conferences as well as meetings with key stakeholders in the results, including cyclists and cycling associations.

## Criteria for inclusion of studies

### Study design

This systematic review protocol includes any study design with the primary focus on injuries and/or illness in competitive cycling disciplines (observational studies, cohort studies, epidemiological studies).

### Sport context

Cycling is an umbrella term used for many individual disciplines (Table 2). Investigation of all types of injuries and/or illnesses sustained during training and/or competition among competitive athletes across all disciplines will be included in this review. All the different cycling disciplines are included in (Table 2). To enable the identification of the most frequently occurring injuries and therein derive injury prevention priorities, studies which report only on a specific type of injury (e.g., stress fractures, concussion, external iliac endo fibrosis), illness (gastrointestinal, respiratory) or body part (e.g., head injuries) will be excluded. Studies in recreational cycling (i.e., not competition-based) will also be excluded

**Table 2:** Description and list of competitive cycling disciplines to be reviewed.

Discipline	Subdiscipline	Summary
Track Cycling	Sprint (SPR)	A short-distance race where cyclists compete head-to-head in a velodrome.
	Team Sprint (TS)	Teams of cyclists compete in a short-distance relay race in a velodrome.
	Keirin (KEI)	Cyclists follow a pacing motorcycle before sprinting to the finish line in a velodrome.
	Individual Pursuit (IP)	Two cyclists start on opposite sides of the track, aiming to get the best time over a distance of 3 km for female athlete and 4 km for males.
	Team Pursuit (TP)	Teams of four cyclists race against the clock and each other over a set distance of 4 km in a velodrome.
	Points Race (PR)	Cyclists earn points for sprints and laps gained in a long-distance race.
	Madison (MAD)	Teams of two cyclists take turns racing and resting in a long-distance race where one cyclist hand slings the other in throughout the race.
	Omnium (OMN)	A multi-race event combining various track cycling disciplines for overall points.
	Scratch Race (SCR)	A straightforward race where the first cyclist to finish wins.
	Time Trial (TT)	Cyclists race alone against the clock over a set distance.
Mountain Biking	Cross-Country Olympic (XCO)	A mass-start race on a hilly, off-road circuit featuring technical sections.
	Cross-Country Marathon (XCM)	A long-distance endurance race on off-road terrain.
	Cross-Country Short Track (XCC)	A shorter, intense version of cross-country racing on a compact circuit.
	Cross-Country Eliminator (XCE)	A knockout format race with heats on a short, technical course.
	Downhill (DHI)	Cyclists race individually downhill on a steep, technical course.
	Enduro (EDR)	A multi-stage race with timed downhill stages and untimed uphill transfers.
	Four-Cross (4X)	Four cyclists race head-to-head on a downhill course with jumps and berms.
	Pump Track	A pump track is a track that consists of rollers and steep turns in various sizes and shapes. The rollers and turns are used to generate speed by pumping the bike, not by pedalling
	Snow Bike	The snow bike is a downhill mountain bike snow event.
	E-Mountain Bike (E-MTB)	Mountain bike racing using electric-assist bicycles.
Road Cycling	Road Race (RR)	A long-distance race on paved roads, typically with a mass start.
	Time Trial (TT)	Cyclists race alone against the clock over a set distance on paved roads.
	Team Time Trial (TTT)	Teams of cyclists race against the clock over a set distance on paved roads.
Trials	20-inch (TR-20)	Cyclists navigate an obstacle course using a bike with 20-inch wheels.
	26-inch (TR-26)	Cyclists navigate an obstacle course using a bike with 26-inch wheels.
Cyclocross	Cyclocross (CX)	Cyclists race on a mixed-terrain course featuring obstacles that may require dismounting.
Gravel Cycling	Gravel Race (GR)	Long-distance races on gravel and mixed-surface roads.
BMX	BMX Racing (BMX-R)	Cyclists race head-to-head on a short, dirt track with jumps and berms.
	BMX Freestyle Park (BMX-FP)	Riders perform tricks and stunts in a skatepark-like environment.
	BMX Freestyle Flatland (BMX-FF)	Riders perform tricks on flat ground without ramps or jumps.
E-Sport	E-Sport Cycling (ES)	Virtual cycling races conducted on online platforms using stationary bikes.
Para-Cycling	Road Race (RR)	Para-cyclists compete in a long-distance race on paved roads.
	Time Trial (TT)	Para-cyclists race alone against the clock over a set distance on paved roads.
	Track Cycling (TC)	Para-cyclists compete in various track events in a velodrome.
	Hand bike (HB)	Cyclists with lower limb impairments race using hand-powered bicycles.
	Tricycle (TRI)	Cyclists with balance impairments race using three-wheeled bicycles.
	Tandem (TAN)	Visually impaired cyclists race on a tandem bike with a sighted pilot.
Indoor Cycling	Cycle Ball (CB)	Teams of two cyclists play a soccer-like game on bicycles.
	Artistic Cycling (AC)	Cyclists perform gymnastic and dance-like routines on fixed-gear bikes.

### Participants

Study inclusion criteria will be framed according to PICO:

• Population (P)- Competitive cyclists. Competitive cyclists are those who are partaking in competition at any level. To partake in any competitive event these cyclists will hold a UCI competition licence. Therefore, all athletes included will hold a UCI racing licence affiliated with their National Governing Body (NGB).
  ○ As this study focused on competitive cyclists, we divided this population into three different levels modified from Heron et al. (10)
    1. An amateur is a cyclist, described as one who practices cycling for non-economic reasons and participates in official domestic competitions.
    2. An elite cyclist is defined as a cyclist who competes at the national/international level but does not receive a regular salary or income for their involvement in the sport.
    3. A Professional Cyclist is defined as a cyclist who competes at the national/international level and receives a regular salary or income for their involvement in the sport.
• The intervention (I) will be classed as injury and/or illness or exposure to injury and or illness. This may be in one event, one month, one competitive season or one year.
• Comparator (C)- Not applicable
• Outcome (O)—The outcome of interest in the studies will be injury/illness type and exposure rate.

### Search methods for identification of studies

A computerised, systematic literature search will be conducted in electronic databases MEDLINE and Embase (both via Ovid), Scopus and Cochrane Library. In line with recommendations for systematic reviews (11) on measurement properties, a hand search of the reference lists of included studies and relevant reviews for additional sources will be conducted. A grey literature search in Google Scholar will also be performed following the database searches.

### Search strategy

The presented search strategy was reviewed and approved by a medical librarian at Queens University Belfast. The search strategies will be based on keywords broken down into those related to cycling and those related to injury and illness (Table 3). Results obtained from the searches will be downloaded in RIS format.

**Table 3:** Keywords used in search strategy.

Cycling Discipline/population Keywords	Injury & Illness Keywords	Medical Subject Headings (MeSH) Terms:
Road cycling	Sports injuries	“Bicycling injuries”
Track cycling	Trauma	“Athletic Injuries”
Mountain biking	Overuse injuries	“Wounds and Injuries”
Cyclocross	Musculoskeletal injuries	
BMX racing	Health surveillance	
Time trial	Illness surveillance	
Gravel cycling	Injury surveillance	
Para-Cycling	Epidemiology	
BMX freestyle		
Artistic cycling		
E-Sport cycling		
Bicycle racing		
Competitive cycling		
Elite cycling		
Professional cycling		

Using Boolean operators (AND, OR), we will combine Cycling and Injury/Illness keywords. For example: (“Competitive cycling” OR “Road cycling”). Using the term “AND” we will combine cycling keywords and injury/illness keywords. Example (“Sports injuries” OR “ Musculoskeletal injuries” AND “Elite Cycling” OR “Competitive cycling”).

### Study selection process

References exported from each database will be imported onto the AI systematic review software “Rayyan” (Cambridge, Massachusetts, USA), where duplicates will be removed and made available for screening. Independent screening by two reviewers will be undertaken in a two-step process: title/abstract screening, followed by full-text review. The reference lists of included articles will be searched to identify any other potentially relevant articles. In addition, citation tracking will also be used to identify potentially eligible studies. Reviewers will apply the predefined inclusion and exclusion criteria to determine study eligibility. A third reviewer will be consulted to resolve disagreements amongst these reviewers and to facilitate consensus. Once study consensus is reached, references will be transferred to the Mendeley reference manager for write-up.

### Data extraction

Systematic extraction of relevant data elements including the following information listed in (Appendix 1). Double verification of extracted data will be completed by the second reviewer to ensure accuracy and completeness. Extracted data will be inputted into a proposed build Microsoft Excel sheet, with all raw data being published within the appendix of the study.

### Methodological quality assessment

Studies will be independently assessed by two reviewers using the National Institute of Health Quality Assessment Tool for Observational Cohort and Cross-Sectional Studies to determine study quality (12) (Appendix 2). The proportion of questions labelled “*yes*” will be calculated with the higher score indicating a lower risk of bias.

### Synthesis of results

Collation of findings from included studies will be done using a narrative approach to present a coherent summary of the evidence. A Tabular/charted presentation of the extracted data will be included where possible. The variables of injuries mainly will be main injury/illness types and definitions, event category, level of participation, diagnostic categories (ie. Medical professionals or self-reported etc), in competition/out of competition and time lost. Statistical methods such as a meta-analysis to combine data if studies are homogenous and comparable. Two systematic reviews have been completed in cycling injury epidemiology, one focused on road cycling (2) and the other on MTB (3). Both reviews were unable to complete any statistical analysis between studies due to the lack of homogeneity between studies. The determination for such an analysis will be at the discretion of the authors, with appropriate statistical input, contingent upon their assessment of discernible dissimilarities among study subpopulations (such as international, elite, and amateur cohorts), exposure types (per 1000hrs, 100rides,100 riders), and reported outcome metrics. This decision-making process aligns with the guidelines advocated by Higgins and Green and Borenstein et al. (13, 14). As per the IOC consensus statement extension for the reporting of injuries and illness in cycling. Meta-analysis of injury and illness rates between disciplines will present rates per 365 athlete days which will allow for comparison of injury and illness rates between cycling disciplines (4).

## Discussion

Injury and illness epidemiology has been identified as a topic of focus for the UCI within their recently published Agenda 2030 (5). This manuscript outlines a protocol to conduct a systematic review of injuries and illness across the different cycling disciplines. The aim of carrying out such research is to present the injury and illness types seen in cycling, their known prevalence, incidences, locations, and burden of injury and illness across the sport of cycling. This systematic review protocol differs from others in injury and illness epidemiology as it will present data from a range of different competitive cycling disciplines. As outlined within the methods and in the IOC consensus statement extension studies with methodological homogeneity will be included within a meta-analysis in which injury rates will be presented per 365 athlete days to allow for comparison between disciplines. In line with the growth of the sport and ensuring that the health and safety of athletes are optimised, the findings of this review will focus on the direction of further high-quality prospective injury and illness studies. From this review, we will aim to gain an understanding of the breadth of injuries and illness across the sport. This will enable researchers to focus future research on areas of most need and align with the 2030 UCI agenda aims to “*promote and support research in cycling epidemiology and medicine, especially for the benefit of lesser-known disciplines”* (5).

Studies have shown that cyclists are not immune to injury or illness (2, 15–19). The range of injuries and illnesses that do occur is vast, however, the reporting of injuries varies significantly with a significant lack of homogeneity between studies and disciplines (2, 4). Injury and Illness surveillance studies in other sports are far more advanced and have seen the development of specific warm-up routines to reduce injury risk such as FIFA 11 (20) in soccer, and Gaelic Athletic Association (GAA) 15 (21) in Gaelic games. Injury and illness surveillance has also been the mainstay in providing evidence that underpins the rule changes to tackle height and reduce head injury risk in professional (22) and amateur (23) rugby. Such examples in other sports highlight the importance of injury surveillance in cycling for medical professionals within teams to adopt the TRIPP model and inform practices that reduce overall injuries and illnesses amongst cyclists. Furthermore, such data on competitive cycling would support the UCI in achieving their agenda 2030 objectives 2(a) “reducing accident risk” and 2 (e) “Implementing independent regulatory medical monitoring”. This review protocol aims to aid research transparency, helping reduce publication bias, preventing selective publication and selective reporting of results. Subsequently, the outcome of this review will provide researchers and sports medicine practitioners with the first step in understanding the current injury and illness surveillance evidence across all cycling disciplines. It will highlight disciplines which lack injury and illness surveillance research in addition to directing the focus for further injury surveillance research amongst the sport.

## Appendix 1: Data Extraction Sheet.

**Table:**

Date		Study 1 Example
Title		
Authors		
Source		
Study Design		
Inclusion criteria		
Exclusion criteria		
Definition of injury		
Definition of illness		
Definition of severity		
Injuries Diagnosed by		
IOC Classification (Y/N)		
Name of event		
UCI Classification		
Length of event (days)		
Distance of events		
Injuries/Illness Excluded		
Comment		
Type of cycling Event:		
Discipline	Sub-Discipline	
Track Cycling	Sprint (SPR)	
	Team Sprint (TS)	
	Keirin (KEI)	
	Individual Pursuit (IP)	
	Team Pursuit (TP)	
	Points Race (PR)	
	Madison (MAD)	
	Omnium (OMN)	
	Scratch Race (SCR)	
	Time Trial (TT)	
Mountain Biking	Cross-Country Olympic (XCO)	
	Cross-Country Marathon (XCM)	
	Cross-Country Short Track (XCC)	
	Cross-Country Eliminator (XCE)	
	Downhill (DHI)	
	Enduro	
	Four-Cross (4X)	
	Pump Track	
	Snow Bike	
	E-Mountain Bike (E-MTB)	
Road Cycling	Road Race (RR)	
	Time Trial (TT)	
	Team Time Trial (TTT)	
Trials	20-inch (TR-20)	
	26-inch (TR-26)	
Cyclocross	Cyclocross (CX)	
Gravel Cycling	Gravel Race (GR)	
BMX	BMX Racing (BMX-R)	
	BMX Freestyle Park (BMX-FP)	
	BMX Freestyle Flatland (BMX-FF)	
E-Sport	E-Sport Cycling (ES)	
Para-Cycling	Road Race (RR)	
	Time Trial (TT)	
	Track Cycling (TC)	
	Hand bike (HB)	
	Tricycle (TRI)	
	Tandem (TAN)	
Indoor Cycling	Cycle Ball (CB)	
	Artistic Cycling (AC)	
Number of Injuries		
Injuries M		
Injuries Fm		
Single Site Injury		
Double Site Injury		
3 Or More		
not listed		
Number of Ilnness		
Neuromuscular Disorder		
Injuries per Incident		
Number of participants M		
Number of participants F		
Total		
Total Exposure		
Days		
Hours		
Racing		
Injury rate (%) M		
Injury rate (%) FM		
Injury rate (%) overall		
illness overall		
Type of injury		
Skin—laceration/abrasion/skin lesion		
Muscle—strain grade 1 or 2		
Ligament—sprain grade 1 or 2		
Muscle—contusion/hematoma		
Muscle—cramps or spasms		
Tendon—tendinopathy/tendinosis/tendinitis		
Joint—arthritis/synovitis/bursitis		
Bone—contusion		
Tendon—fasciitis/aponeurosis injury		
Joint—dislocation/subluxation/instability		
Other injury (assessment note required)		
Bone—acute fracture		
Neuro—concussion		
Joint—lesion of meniscus or cartilage		
Joint—impingement		
Tendon—sprain grade 1 or 2		
n/a		
Ligament—rupture grade 3		
Muscle—rupture grade 3		
Bone—stress fracture/stress reaction		
Neuro—peripheral nerve injury		
Tendon—rupture grade 3		
Neuro—spinal cord injury		
Injury without tissue type specified		
Area of injury		
Pelvis		
Hip		
Anterior thigh		
Posterior thigh		
Knee		
Anterior lower leg		
Posterior lower leg		
Calf/Achilles		
Foot/toes		
Ankle		
Shoulder		
Upper arm		
Elbow		
Forearm		
Wrist		
Hand/fingers		
Thumb		
Face (including eyes, ears, nose)		
Head		
Neck		
Chest		
Abdomen		
Thoracic		
Upper back		
Lower back		
Back		
Multiple body locations		
Dental		
n/a		
Upper extremity		
Lower		
Head/neck		
Torso/back		
Multiple sites		
Combined limb		
Able to continue cycling: Yes (%)		
Able to continue cycling: No		
Severity of injuries Overall		
Severity of injuries M		
Severity of injuries FM		
Types of Illness		
Dermatologic		
Thermoregulatory		
Respiratory/ear, nose, throat		
Gastro-intestinal		
Other		
Uro-genital/gynaecological		
Neurological/psychiatric		
Cardiovascular		
Dental		
Allergic/immunological		
Musculoskeletal		
Metabolic/endocrinological		
Haematological		
Aetiology		
Allergic		
Environmental–exercise-related		
Environmental—non-exercise		
Immunological/inflammatory		
Infection		
Neoplasm		
Metabolic/nutritional		
Thrombotic/haemorrhagic		
Degenerative or chronic condition		
Developmental anomaly		
Drug-related/poisoning		
Multiple		
n/a		
Unknown, or not specified		
Contributing factors reported?		
Acute (%)		
Chronic (%)		
Contact (with another athlete)		
Non-contact trauma		
Overuse (sudden onset)		
Overuse (gradual onset)		
Contact (with stagnant object)		
Contact (with moving object)		
Recurrence of previous injury		
Other cause not otherwise specified		
Other		
n/a		
Field of Play conditions		
Equipment failure		
Weather conditions		
Violation of rules		
Rider related fault		

## Appendix 2: National Institute of Health Quality Assessment Tool for Observational Cohort and Cross-Sectional Studies to determine study quality.

**Table:**

Criteria
1. Was the research question or objective in this paper clearly stated?
2. Was the study population clearly specified and defined?
3. Was the participation rate of eligible persons at least 50%?
4. Were all the subjects selected or recruited from the same or similar populations (including the same time period)? Were inclusion and exclusion criteria for being in the study prespecified and applied uniformly to all participants?
5. Was a sample size justification, power description, or variance and effect estimates provided?
6. For the analyses in this paper, were the exposure(s) of interest measured prior to the outcome(s) being measured?
7. Was the timeframe sufficient so that one could reasonably expect to see an association between exposure and outcome if it existed?
8. For exposures that can vary in amount or level, did the study examine different levels of the exposure as related to the outcome (e.g., categories of exposure, or exposure measured as continuous variable)?
9. Were the exposure measures (independent variables) clearly defined, valid, reliable, and implemented consistently across all study participants?
10. Was the exposure(s) assessed more than once over time?
11. Were the outcome measures (dependent variables) clearly defined, valid, reliable, and implemented consistently across all study participants?
12. Were the outcome assessors blinded to the exposure status of participants?
13. Was loss to follow-up after baseline 20% or less?
14. Were key potential confounding variables measured and adjusted statistically for their impact on the relationship between exposure(s) and outcome(s)?
Total percentage of applicable questions answered “Y”

*Y, Yes; N, No; N/A, not applicable.

## References

[1] UCI. Spectacular TV and Digital Audiences for 2023 UCI Cycling World Championships in Glasgow and Across Scotland (2023). Available online at: https://www.uci.org/pressrelease/spectacular-tv-and-digital-audiences-for-2023-uci-cycling-world/3KSV2mdsYiRRoPBupy1tDT (cited December 8, 2023).

[2] Rooney D Sarriegui I Heron N. ‘As easy as riding a bike’: a systematic review of injuries and illness in road cycling. BMJ Open Sport Exerc Med. (2020) 6(1):e000840. 10.1136/bmjsem-2020-000840 34422283 PMC8323466

[3] Buchholtz K Lambert M Corten L Burgess TL. Incidence of injuries, illness and related risk factors in cross-country marathon mountain biking events: a systematic search and review. Sports Med Open. (2021) 7(1):68. 10.1186/s40798-021-00357-z 34564784 PMC8464637

[4] Clarsen B Pluim BM Moreno-Pérez V Bigard X Blauwet C Del Coso J Methods for epidemiological studies in competitive cycling: an extension of the IOC consensus statement on methods for recording and reporting of epidemiological data on injury and illness in sport 2020. Br J Sports Med. (2021) 55(22):1262–9. 10.1136/bjsports-2020-103906 33980546

[5] Union Cycliste Internationale. Agenda 2030 (2023). Available online at: https://assets.ctfassets.net/761l7gh5x5an/6RrOHtU0QlyN80MDJ7vJm3/cf54c913960a66a71baaac379ef12b88/2022_UCI_AGENDA2030_web_EN.pdf (cited December 8, 2023).

[6] van Mechelen W Hlobil H Kemper HCG. Incidence, severity, aetiology and prevention of sports injuries. Sports Med. (1992) 14(2):82–99. 10.2165/00007256-199214020-00002 1509229

[7] Finch C. A new framework for research leading to sports injury prevention. J Sci Med Sport. (2006) 9(1–2):3–9. 10.1016/j.jsams.2006.02.009 16616614

[8] Page MJ McKenzie JE Bossuyt PM Boutron I Hoffmann TC Mulrow CD The PRISMA 2020 statement: an updated guideline for reporting systematic reviews. BMJ. (2021) 372:n71. 10.1136/bmj.n71 33782057 PMC8005924

[9] Ardern CL Büttner F Andrade R Weir A Ashe MC Holden S Implementing the 27 PRISMA 2020 statement items for systematic reviews in the sport and exercise medicine, musculoskeletal rehabilitation and sports science fields: the PERSiST (implementing prisma in exercise, rehabilitation, sport medicine and SporTs science) guidance. Br J Sports Med. (2022) 56(4):175–95. 10.1136/bjsports-2021-103987 34625401 PMC8862073

[10] Heron N Sarriegui I Jones N Nolan R. International consensus statement on injury and illness reporting in professional road cycling. Phys Sportsmed. (2021) 49(2):130–6. 10.1080/00913847.2020.1830692 33000984

[11] Hopewell S Clarke M Lefebvre C Scherer R. Handsearching versus electronic searching to identify reports of randomized trials. In: Hopewell S, editor. Cochrane Database of Systematic Reviews. Chichester, UK: John Wiley & Sons, Ltd (2002). 10.1002/14651858.MR000001.pub2 PMC7437388 17443625

[12] National Institute of Health. National Institute of Health Quality Assessment Tool for Observational Cohort and Cross-Sectional Studies (2017). Available online at: https://www.nhlbi.nih.gov/health-topics/study-quality-assessment-tools (cited March 28, 2024).

[13] Higgins J Green S. Cochrane Handbook for Systematic Reviews of Interventions. Wiley (2008).

[14] Borenstein M Hedges LV Higgins JPT Rothstein HR. Introduction to Meta-Analysis. Wiley (2009).

[15] Willick SE Ehn M Teramoto M Klatt JWB Finnoff JT Saad K The national interscholastic cycling association mountain biking injury surveillance system: 40,000 student-athlete-years of data. Curr Sports Med Rep. (2021) 20(6):291–7. 10.1249/JSR.0000000000000850 34099606 PMC8710302

[16] Kim PTW Jangra D Ritchie AH Lower ME Kasic S Brown DR Mountain billing injuries requiring trauma center admission: a 10-year regional trauma system experience. J Trauma Injury Infection Crit Care. (2006) 60(2):312–8. 10.1097/01.ta.0000202714.31780.5f 16508488

[17] Ehn M Teramoto M Cushman DM Saad K Willick S. The national interscholastic cycling association (nica) mountain biking injury surveillance system (iss): analysis of 66,588 student athlete-years of injury data. Int J Environ Res Public Health. (2021) 18(11). 34072534 10.3390/ijerph18115856 PMC8198101

[18] Bigdon SF Hecht V Fairhurst PG Deml MC Exadaktylos AK Albers CE. Injuries in alpine summer sports - types, frequency and prevention: a systematic review. BMC Sports Sci Med Rehabil. (2022) 14(1):79. 10.1186/s13102-022-00468-4 35501847 PMC9063189

[19] Braybrook PJ Tohira H Birnie T Brink D Finn J Buzzacott P. Types and anatomical locations of injuries among mountain bikers and hikers: a systematic review. PLoS One. (2023) 18(8):e0285614. 10.1371/journal.pone.0285614 37647303 PMC10468092

[20] Magoshi H Hoshiba T Tohyama M Hirose N Fukubayashi T. Effect of the FIFA 11+ injury prevention program in collegiate female football players over three consecutive seasons. Scand J Med Sci Sports. (2023) 33(8):1494–508. 10.1111/sms.14379 37211876

[21] Schlingermann BE Lodge CA Gissane C Rankin PM. Effects of the gaelic athletic association 15 on lower extremity injury incidence and neuromuscular functional outcomes in collegiate gaelic games. J Strength Cond Res. (2018) 32(7):1993–2001. 10.1519/JSC.0000000000002108 28817505

[22] Tucker R Raftery M Kemp S Brown J Fuller G Hester B Risk factors for head injury events in professional rugby union: a video analysis of 464 head injury events to inform proposed injury prevention strategies. Br J Sports Med. (2017) 51(15):1152–7. 10.1136/bjsports-2017-097895 28642222

[23] van Tonder R Hendricks S Starling L Surmon S Viviers P Kraak W Tackling the tackle 1: a descriptive analysis of 14,679 tackles and risk factors for high tackles in a community-level male amateur rugby union competition during a lowered tackle height law variation trial. J Sci Med Sport. (2024) 27(1):57–62. 10.1016/j.jsams.2023.10.011 37932203

